# Utility of indocyanine green fluorescent dye in emergency general surgery: a review of the contemporary literature

**DOI:** 10.3389/fsurg.2024.1345831

**Published:** 2024-02-14

**Authors:** Mohamed H. Khalaf, Husham Abdelrahman, Ayman El-Menyar, Ibrahim Afifi, Ahmad Kloub, Ammar Al-Hassani, Sandro Rizoli, Hassan Al-Thani

**Affiliations:** ^1^ Department of Surgery, Hamad Medical Corporation, Doha, Qatar; ^2^ Department of Surgery, Trauma Surgery, Hamad Medical Corporation, Doha, Qatar; ^3^ Clinical Research, Trauma & Vascular Surgery Section, Hamad Medical Corporation, Doha, Qatar; ^4^ Department of Clinical Medicine, Weill Cornell Medicine, Doha, Qatar

**Keywords:** indocyanine green (ICG), fluorescent, acute care, emergency general surgery, surgery, perfusion

## Abstract

For decades, indocyanine green (ICG) has been available for medical and surgical use. The indications for ICG use in surgery have expanded where guided surgery directed by fluorescence and near-infrared fluorescent imaging offers numerous advantages. Recently, surgeons have reported using ICG operative navigation in the emergency setting, with fluorescent cholangiography being the most common procedure. The utility of ICG also involves real-time perfusion assessment, such as ischemic organs and limbs. The rising use of ICG in surgery can be explained by the ICG's rapid technological evolution, accuracy, ease of use, and great potential to guide precision surgical diagnosis and management. The review aims to summarize the current literature on the uses of ICG in emergency general surgery. It provides a comprehensive and practical summary of the use of ICG, including indication, route of administration, and dosages. To simplify the application of ICG, we subdivided its use into anatomical mapping and perfusion assessment. Anatomical mapping includes the biliary tree, ureters, and bowel. Perfusion assessment includes bowel, pancreas, skin and soft tissue, and gonads. This review provides a reference to emergency general surgeons to aid in implementing ICG in the emergency setting for more enhanced and safer patient care.

## 1 Introduction

Indocyanine green (ICG) is a water-soluble fluorescent dye used in numerous clinical settings for decades. ICG was developed in 1955 by Kodak Research Laboratories and was approved for human use by the FDA in 1959. Since then, ICG has had clinical applications including, but not limited to, assessing cardiac output, hepatic metabolism, and retinal function (1–3). More recently, with the development of fluorescent imaging, ICG use has expanded into surgery. ICG is currently used in vascular, general, colorectal, hepatobiliary, breast, gynecological, plastic, and urological surgery.

ICG is available in powder form at a dose of 25 mg. It comes with 10 ml of sterile water to dissolve the powder. Following reconstitution, a 25 mg vial of ICG contains 2.5 mg of dye per 1 ml (4). After intravenous injection, 98% is bound plasma proteins; the remaining is free in the serum (1). The solution formed has partial stability and should be used within 6 h after dilution (5).

ICG is exclusively metabolized by the liver and excreted in the bile. ICG has a short half-life of 3-4 min (3). Thus, for ICG angiography, the near-infrared fluorescent imaging (NIFI) device must be set and ready before the intravenous injection of ICG for optimal imaging. For example, in trauma surgery, ICG has been used with repeated IV boluses for assessment of bowel perfusion before and after bowel anastomosis (6).

ICG has a proven safety record with doses between 0.1 and 0.5 mg/kg, with relative contraindications being iodine or shellfish allergies. Rare reports of anaphylactic reactions have been reported, which are mainly attributed to high doses (7, 8). In surgery, ICG has become popular recently due to the easy setup of ICG dye and NIFI camera (or scope). Depending on the intended ICG use, there are different methods of administration. The review aims to summarize the use of ICG in emergency general surgery, classifying its use into anatomical mapping and perfusion assessment. This classification is based on our interpretation of the reviewed literature to help simplify its usage for surgeons. This review did not discuss, in detail, the utility of ICG in the surgical management of traumatic injury, as this was extensively described in prior works (6, 9).

### 1.1 Search method

A literature search was performed on both PubMed and Medline. The search terms included (Indocyanine green acute surgery) OR (Indocyanine green emergency surgery) OR (indocyanine green urgent surgery)) OR (ICG acute surgery)) OR (ICG emergency surgery)) OR (ICG urgent surgery)). Two authors (MHK and HA) reviewed publications from 1950 to April 2023, which were limited to publications in English and included only human subjects. Case reports and video abstracts were also included.

**Figure 1:**
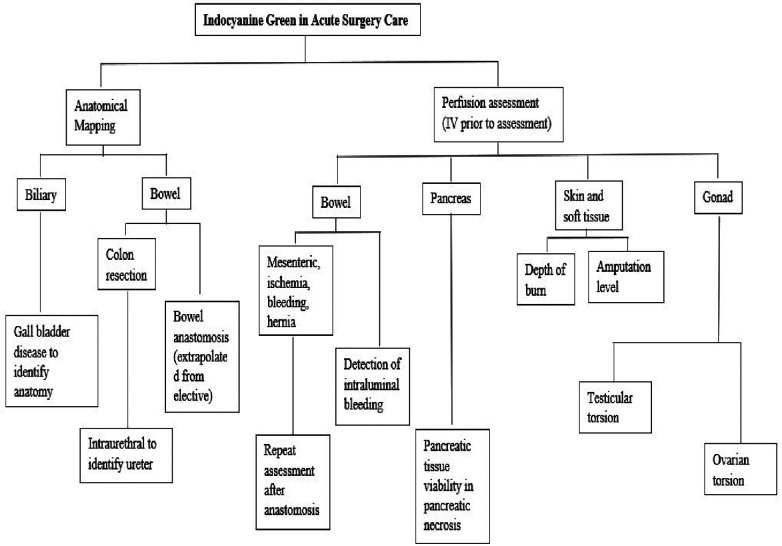
Flowchart of the review.

Further review was carried out in each area of interest for additional background. No statistical analysis was performed. Figure 1 shows the flowchart of the utility of ICG in the present review.

## 2 Results & discussion

### 2.1 Anatomical mapping

#### 2.1.1 Gallbladder surgery

Laparoscopic cholecystectomy (LC) is a commonly performed surgical procedure. LC is the standard technique for treating benign gallbladder disease in elective and acute settings (10, 11). LC has been proven to be advantageous over open cholecystectomy (12). The critical view of safety (CVS) was a suggested approach to avoid iatrogenic bile duct injury (BDI) (13). Bile duct injury is a well-documented complication of cholecystectomy. The emergence of LC was associated with an increased incidence of BDI (12). Nevertheless, iatrogenic BDI occurs at a rate of 0.08% up to 1.5% (14). Surgeon experience, anatomical variation, and severity of inflammation are factors that may affect the occurrence of BDI (15–18). BDI impacts patients**’** quality of life and survival (14, 19, 20).

Intraoperative cholangiography (IOC) is a well-established modality of imaging that can be used to reduce BDI incidence. During a cholecystectomy, IOC may aid in delineating the biliary tree anatomy. However, its routine use is debatable (21). Standard IOC involves the identification and partial opening of the cystic duct, insertion of a catheter, followed by the injection of radio-opaque contrast material. The x-ray portable C-arm with a sterile cover is positioned over the patient while the contrast is injected. Intraoperative cholangiography is fraught with technical difficulties, which may prevent appropriate identification of the relevant anatomy. The dissection of the cystic duct to perform the IOC can cause a BDI; thus, less invasive, and safer methods are warranted.

Recently, ICG near-infrared (NI) fluorescent cholangiography (FC) has been shown to be a potential alternative with many advantages over contrast IOC. As opposed to IOC, FC is not as invasive and does not require cystic duct dissection, transection, or cannulation. FC does not require disruption of the surgery or exposure of the patient to radiation since no x-ray equipment is needed. ICG with near-infrared fluorescence cholangiography has been reported as an adjunct technique in identifying biliary anatomy in the elective setting (22–24).

Furthermore, the surgeon can visualize the biliary tree anatomy directly instead of indirectly via x-ray IOC. ICG can also be efficiently utilized during robotic cholecystectomy (RC) using the DaVinci robot with Firefly capability and easy toggling in and out of fluorescence mode during surgery (24–26).

**Figure 2:**
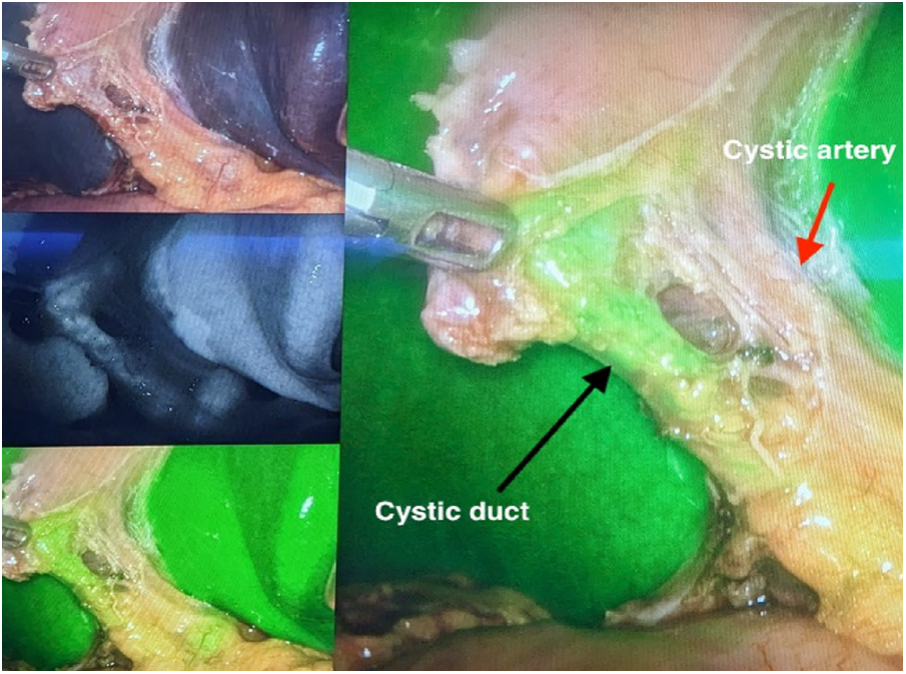
The use of ICG in GB surgery.

**Figure 3:**
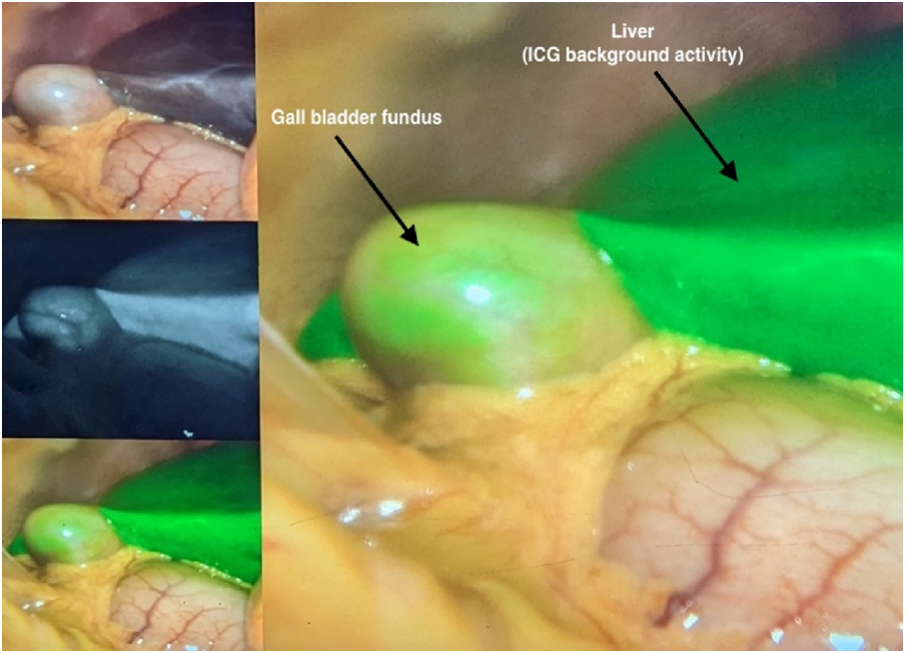
The use of ICG in GB surgery.

An additional advantage of ICG cholangiography is its cost-effectiveness. Even though the initial FC setup may appear more expensive, Reeves et al. demonstrated a potential cost saving over the long term due to shorter operative times and a lower conversion rate to open surgeries (27). Compared to intraoperative cholangiography, Dip et al. also showed that in a single-center study, FC provided a cost-benefit compared to IOC in elective and acute cholecystitis cases (28). (Figures 2,3: examples of the use of ICG in GB surgery).

ICG NIFC involves either intravenous (IV) or intra-biliary (IB) injection of ICG. IB injection can be done by direct injection into the gallbladder or via a gallbladder/biliary drainage tube (cholecystostomy). The IB injection into the gallbladder often provides a clearer image of the biliary anatomy due to reduced hepatic background activity compared to IV ICG administration (29–31). ICG is excreted exclusively in the bile, and specialized cameras can detect it because it emits light when bound to proteins. NI fluorescent capable laparoscopes are used to visualize ICG and can allow for the identification of the extrahepatic biliary anatomy (32, 33).

IB injection of ICG directly into the gallbladder is a simple and effective way to identify biliary tree anatomy in cases of severe cholecystitis, particularly when the gallbladder is distended, tense, and challenging to grasp. In such cases, the operating surgeon may drain part of the gallbladder, followed by injection of ICG into the gallbladder. The reduced background signal from the liver makes it easier to visualize pertinent structures while dissecting the triangle of Calot (29–31). IB may be superior to the IV route, though no study has tested this proposition.

In cases where an intravenous approach is chosen, different doses of ICG have been reported in other studies. In a recent Delphi survey, the consensus was weight-based doses of 0.05 mg/kg as a minimum. Intravenous injection is administered at least 30 min before the surgery (34). Conversely, direct IB injection does not require pre-administration. However, there is no specific dose recommended for IB administration. In reports where IB was used, varying amounts were injected with an average of 5 ml (ICG 25 mg diluted in 10 ml sterile water) reported as sufficient (30, 31). Adverse reactions such as hypotension, urticaria, and shock have been reported with the administration of higher doses of ICG (0.5 mg/kg) (8). Administration can be repeated while staying within a reasonable range of up to 0.25 mg/kg.

#### 2.1.2 Indications

In the elective setting, ICG FC is faster than IOC and has a higher visualization rate of the biliary anatomy (22). Failed intraoperative cholangiography is expected due to technical difficulties. ICG FC can identify the cystic duct in up to 60% without dissection and 95% after dissection. Conversely, IOC has the advantage of identifying the common right and left hepatic ducts and choledocholithiasis. FC was used to identify the cystic duct in 80% of the patients where intraoperative cholangiography was not achievable (22). Arguably, if ICG delineates the biliary anatomy, it can make the surgical dissection safer. This observation is supported by Keeratibharat et al. who achieved similar initial ICG FC visualization rates before dissection and Ahmed, et al. who achieved 100% detection rates regardless of cholecystitis grade (23, 35). Hitwashi.et al. reported lower ICG cystic duct detection rates in acute cholecystitis and a higher conversion to open when the cystic duct is not detected (36).

The Tokyo Guidelines 2018 recommend early cholecystectomy for acute cholecystitis. However, there was no consensus on the use of ICG during technically challenging LC with a risk of BDI. The guidelines suggested ICG as a possible adjunct without making any firm recommendation (37). In a recent meta-analysis adjusting for both acute and chronic cholecystitis, Dip et al. concluded that using ICG and near-infrared fluorescent cholangiography is associated with reduced rates of BDI and conversion to open cholecystectomy (38).

Furthermore, Broderick et al. in the largest single center series, demonstrated in their patient population with acute cholecystitis, that ICG use was associated with shorter operative times, lower rates of bail-out procedures and shorter hospital length of stay. There was no difference in outcomes regarding bile duct injury (39). Results from Di Maggio et al. showed reduced operative times as well but no difference in conversion rates (40). Quaresima et al. also reported shorter operative times for ICG FC use than conventional IOC (41). However, FC has limitations, including an inability to identify stones in the extrahepatic biliary tree. Thus, FC should be reserved solely to assist in defining the anatomy.

#### 2.1.3 Organ identification

A randomized trial by Dip et al. compared anatomical landmark identification during conventional white light cholecystectomy to fluorescent cholangiography and concluded that pre dissection anatomical identification rates were higher in the fluorescent cholangiography arm. These data included around 14% of patients undergoing urgent/emergent cholecystectomies. Two bile duct injuries occurred in the conventional white light cholecystectomy group but did not reach statistical significance to conclude that ICG was associated with improved outcomes (42). A randomized control trial by She, et al. concluded no difference in outcomes between conventional cholecystectomy compared to an ICG group in the emergency setting (43). Turcotte et al. reported no change in operative times or the need for bail-out procedures in patients where ICG was used in urgent LC (44).

Compared to standard LC with no imaging, van Dam et al. found that ICG fluorescent imaging proved to facilitate faster identification of the cystic duct and more frequent identification of the common bile duct. Only one BDI occurred, and ICG successfully identified the injury (45).

Other reports experienced lower cystic duct detection rates with acute inflammation while using ICG imaging. Bandari, et al. reported lower rates of CBD and cystic duct detection in patients with acute cholecystitis at 80% and 90% respectively compared to 100% visualization in other groups (cholelithiasis, chronic cholecystitis, and mucocele, polyps) (46).

#### 2.1.4 Additional applications

Further uses of ICG in rare cases such as gallbladder volvulus, completion cholecystectomy, and gallbladder agenesis are to help identify the anatomy (47–49). A report from Okada et al. described ICG injection through a naso-biliary transduodenal gallbladder drainage tube during interval elective cholecystectomy. The injection of ICG assisted them in the identification of a cholecystoduodenal fistula, which was successfully managed (50). Additionally, Yoshiya et al. reported ICG injection through a percutaneous transhepatic gallbladder drainage tube to help identify the anatomy (51).

Fluorescent cholangiography offers promising potential for use in LC and RC. Its ease of use and accessibility, once established in a facility, is encouraging to emergency general surgeons. A review of published literature regarding the use of ICG in an emergency setting shows that there still needs to be a definitive recommendation supporting the routine use of ICG in laparoscopic cholecystectomies. It has become clear that ICG is a useful, promising agent that can help the surgeon identify the biliary anatomy when there is ongoing severe inflammation or variant anatomy. First impressions are important when interpreting biliary anatomy. The misidentification of the common bile duct as the cystic duct is a misperception with detrimental effects on the patient (52, 53). Since ICG can help mitigate the risk of BDI with high rates of cystic duct identification with no dissection and the percentage of identified cystic ducts goes up with dissection, we advocate its use. Whether its use will become the standard of care in cholecystectomies is yet to be seen. Additionally, ICG FC can be used as an educational tool to aid medical students and residents in anatomical identification education (54, 55).

It does provide a navigational advantage to identify the critical view of safety in cases with difficult-to-delineate anatomy. FC can improve outcomes at a reduced cost and a more efficient rate. Further prospective large studies are required before standardization of care focusing on the incidence of BDI, time to visualization of biliary anatomy, and outcomes when ICG fails to help identify. A Summary of literature pertaining to the utility of ICG for anatomical mapping is provided in Table 1 (24, 29–32, 35, 36, 39–44, 46–51, 56–61).

**Table 1:** Summary of literature pertaining to anatomical mapping using ICG.

Authors	Target organ	Patients *N*	Emergency general patients *N*	Dose or concentration	Route of administration
Di Maggio, et al. (40)	GB	57	33	0.25 mg	IV
Castagneto-Gissey, et al. (29)	GB	18	15	0.01 mg/kg	IV
Castagneto-Gissey, et al. (29)	GB	17	14	5 ml	IB
She, et al. (43)	GB	46	46	–	–
Ahmed, et al. (35)	GB	70	12	5 mg	IV
Brito-Carmona, et al. (56)	GB	1	1	1 ml ICG in 10 NS	IB
Okada, et al. (50)	GB	1	1	0.025 mg/ml	IB
Utsunomiya, et al. (49)	GB	2	2	0.25 mg/kg	IV
Broderick, et al. (39)	GB	1,389	137	7.5 mg	IV
Bandari, et al. (46)	GB	43	10	5 mg	IV
Gangemi, et al. (26)	GB	965	225	2.5mg	IV
Daskalaki, et al. (25)	GB	212	28	2.5mg	IV
Turcotte, et al. (44)	GB	198	105	2.5 mg	IV
Elzubeir, et al. (48)	Biliary tree	1	1	NR	IV
Agnus, et al. (57)	GB	314	58	0.28 mg/kg	IV
Nitta, et al. (30)	GB	1	1	0.025 mg/ml	IB
Quaresima, et al. (41)	GB	88	21	IV 0.1 mg/kg/IB 2 mg	IV/IB
Jao, et al. (31)	GB	2	2	12.5 mg	IB
Jao,et al. (31)	GB	2	2	2.5 mg	IV
Dip, et al. (42)	GB	639	88	0.05 mg/kg	IV
Bustos, et al. (47)	GB	1	1	NR	IV
Yoshiya, et al. (51)	GB	130	31	2.5 mg × 2	IV
Tsutsui, et al. (58)	GB	1	1	12.5 mg	IV
Sharma, et al. (24)	GB	287	64	NR	IV
Hiwatashi, et al. (36)	GB	65	32	2.5 mg	IV
Ishizawa, et al. (32)	GB	52	2	2.5 mg	IV
Rodríguez-Zentner, et al. (59)	Ureter	30	18	–	IU
Soriano, et al. (60)	Ureter	83	31	5 ml/ureter	IU
White, et al. (61)	Ureter	16	7	5 ml/ureter	IU
GB: gallbladder, IV: intravenous, IB: intra-biliary, IU: intra-ureteric, NR: not reported.					

## 3 Colon surgery

Colon resections can be performed in the emergency general surgery as a treatment for diverticular disease where there is potential for ICG utilization (60–62). Injury to surrounding structures, including the ureters, is a potential complication. In colon resections, ureteral injuries are a serious complication, with an incidence reaching up to 5% (63). Ureteric stenting has been used to help with ureteric identification, injury recognition, and repair (64). It has been shown that pre-procedural stent placement in colorectal resections does not affect intraoperative recognition of iatrogenic ureteric injury (65). ICG can be used to visualize the ureters clearly. ICG is useful in cancer surgery and has shown to be beneficial in colon resection for diverticular disease, where inflammation can distort the visualization of the ureters (60–62, 66–68). In elective surgery, ICG has also been used to detect anastomotic leaks using an intraluminal injection of diluted dye. This can be used in left-sided and rectal anastomoses through rectal intra-luminal injection (69).

ICG administration is in the operating room, after induction of anesthesia, utilizing cystoscopy. Retrograde ureteric catheterization is performed, and ICG is injected into the ureter with or without placement of a ureteric catheter. This injection of ICG is performed once at the start of the procedure and lasts throughout the resection with adequate visualization of the ureters. A ureteral catheter is usually left in place as well and removed at the end of surgery, with some reports of self-resolving hematuria after surgery (68). The intra-ureteric (IU) ICG is detected using NIFI (61). More recently, Soriano, et al. reported that intra-ureteric ICG injection without ureteral catheterization is less time consuming and is just as efficient with no effect on visualization time (60).

The exact dose used by various reports varies. Doses vary between 1 and 2 ml of ICG (2.5 mg/ml) diluted in 5 ml of distilled water injected into each ureter. This can also be followed by a 10cc flush of distilled water (67, 68, 70). This is sufficient for ureteric identification throughout the surgery.

Ureteral identification in diverticulitis patients is pertinent in the emergency general surgery population. Patients with diverticulitis planned for laparoscopic or robotic colonic resections would benefit from using ICG to identify ureters due to colon resection. It can be helpful in cases with complicated diverticulitis such as abscess, fistula, or stricture (70).

Ureteric identification using ICG achieves success rates of up to 99%. The right ureter is identified quickly at the start of surgery in about a minute. Right ureter visualization can be slowed down in obese patients (60). The left ureter can take more time to visualize, requiring initial medial to lateral dissection before the ureter is seen. Continued identification has been reported to last up to eight hours throughout the reported cases (59, 60, 67, 68, 71).

In summary, ICG is promising for ureteric identification in colon resections. It does require IU ICG injection, which relies on the presence of a urologist to perform this at the start of the procedure. IU ICG injection typically entails ureteric stenting. Initial reports of ICG IU use without stenting are positive and possibly negate the need for stent placement, thereby avoiding stenting complications. While no guidelines support the routine use of ICG in colon resections in patients with diverticulitis, on review of the reported data without ureteric stenting, ICG is a helpful adjunct and a continuous intraoperative guide to ureteric identification. Further studies are required on larger cohorts with a focus on the emergency surgery setting and the benefit of routine ICG use for ureteric identification in patients with obstruction and perforated diverticulitis.

### 3.1 Perfusion assessment

#### 3.1.1 Bowel

**Figure 4:**
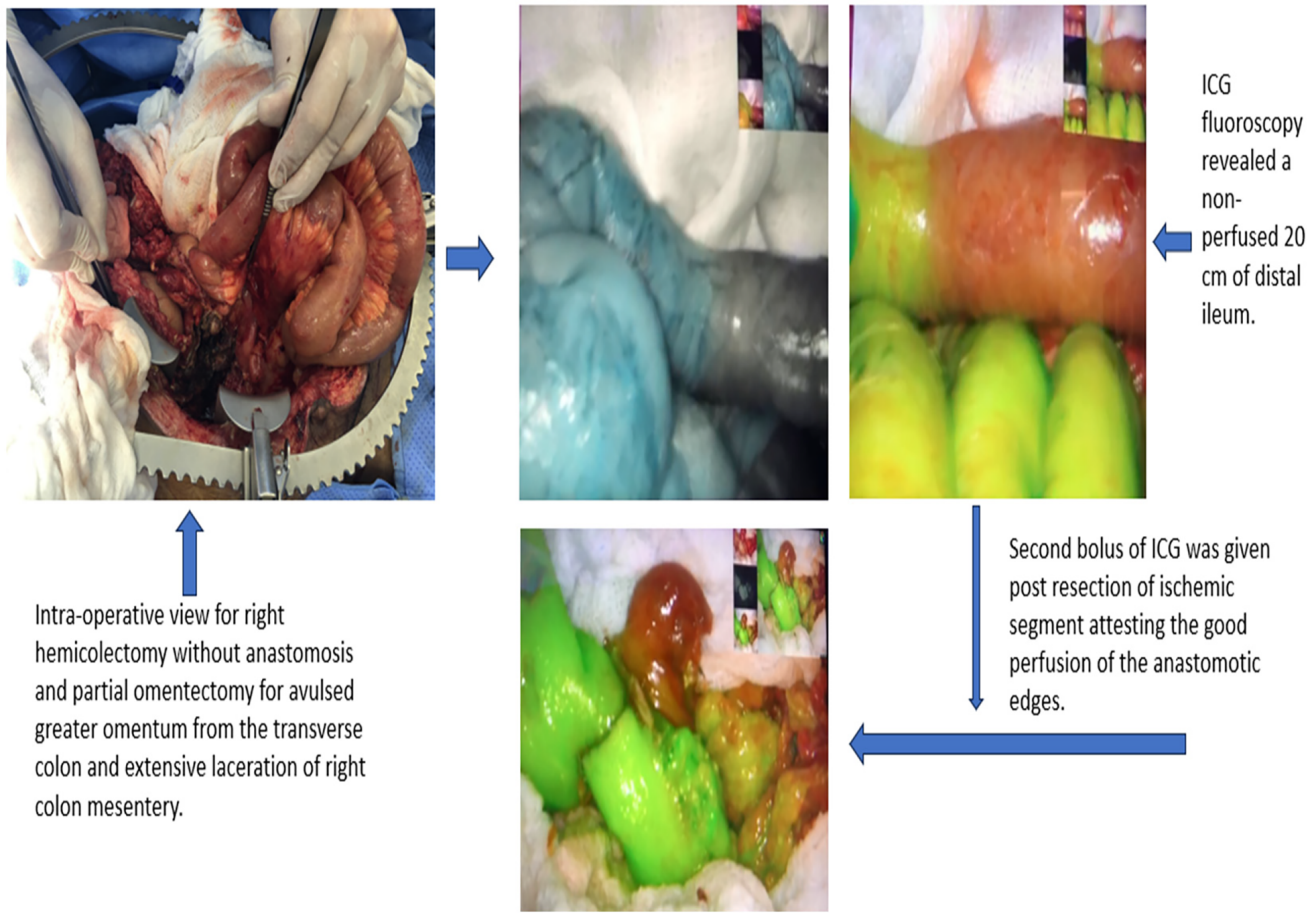
Example of ICG use for bowel ischemia/perfusion.

Acute mesenteric ischemia (AMI) and bowel obstruction are gastrointestinal pathologies that face emergency general surgeons with varying incidences. They can present with varying degrees of severity and can lead to devastating sequelae if left untreated (Figure 4 shows an example of ICG use for bowel ischemia/perfusion).

Acute mesenteric ischemia (AMI) has a low incidence of 0.1%–0.2% of admissions to the emergency department and is an uncommon cause of abdominal pain (72–74). AMI can be classified into non-occlusive or occlusive disease. Occlusive mesenteric ischemia can be caused by arterial embolism, thrombosis, or venous thrombosis (75, 76). Bowel obstruction and perforation may be caused by benign or malignant disease and will sometimes necessitate surgical exploration and bowel resection.

ICG fluorescence angiography has been studied in elective and emergency care and open and laparoscopic surgery to assess anastomotic bowel perfusion effectively. It is shown to have favorable results in a reduction of post-surgical anastomotic leaks in cancer surgery (77, 78).

Typically, the administration of ICG will be through the intravenous route. An additional reported method of administration is through interventional radiology positioned catheter near the site of bowel bleeding, which will be discussed in detail below.

ICG can be administered intravenously at a dose of 5–7.5 mg or 2–3 ml (2.5 mg/ml), followed by a flush of normal saline. It will take about 2–3 min to appear using NIFI.

#### 3.1.2 Indications

The diagnosis of AMI involves a high index of clinical suspicion and laboratory and radiological investigation. Computed tomography is the standard of diagnosis. When the diagnosis is established and there is suspicion of bowel ischemia, surgical exploration is warranted (79). Surgical exploration may be laparoscopic or open exploratory laparotomy to assess bowel viability. Laparoscopic exploration is done to assess bowel status depending on the subjective interpretation. Bowel serosa surface color, pulsation, and bleeding are used to assess perfusion. Laparoscopic exploration with ICG FA can be used to assess perfusion when in doubt and offers a more subjective approach.

Revascularization is a goal of treatment in embolic cases of AMI. ICG angiography can be implemented in the setting of AMI to assess bowel perfusion. Initial case reports outlined the feasibility of using ICG to detect ischemic bowel segments. Alemanno et al. used ICG in a patient who had previously undergone endovascular aortic repair of a type B aortic dissection and complained of abdominal pain. The CT was inconclusive, but the symptoms progressed. In this setting, ICG was used in the initial laparoscopic exploration to confirm the diagnosis and then again on laparotomy to guide the resection of the ischemic bowel (80). Furusawa et al. used ICG in the setting of AMI with no gross evidence of ischemia. The decision not to resect was supported by adequate perfusion on ICG FA (81).

Non-occlusive mesenteric ischemia (NOMI) is a more challenging diagnosis due to nonspecific symptoms and findings on imaging. Ishiyama et al. reported 40 patients who underwent surgical exploration for NOMI. Fifteen of those patients received ICG to evaluate intestinal perfusion. There was no difference in outcomes regarding reoperation related to intestinal necrosis or 30-day mortality. ICG FA changed the operation in a major way in three patients and had them revise the resection line in six patients (82). In a case report by Nitori et al. ICG FA aided the decision in avoiding a jejunal resection with no post-operative adverse outcome in a patient with NOMI (83). Small mesenteric venous thrombosis can also result in bowel ischemia, and ICG has been reported to help delineate the resection line in a patient with venous thrombosis (84).

#### 3.1.3 Other indications of ICG FA in gastrointestinal diseases

In pediatric emergency general surgery, a case report by Iinuma et al. reported using ICG as prognostic indicator of small bowel structuring disease. Following bowel resection due to volvulus, FA showed an area of decreased intensity and abnormal vascular flow. The area appeared grossly viable and persevered. The patient then developed a partial structure at the area of questionable fluorescent perfusion and required additional resection (85). Similarly, in an adult, a case report details an area of questionable ICG perfusion in an initial exploration, which was managed conservatively and required resection 24 days later (86).

**Figure 5:**
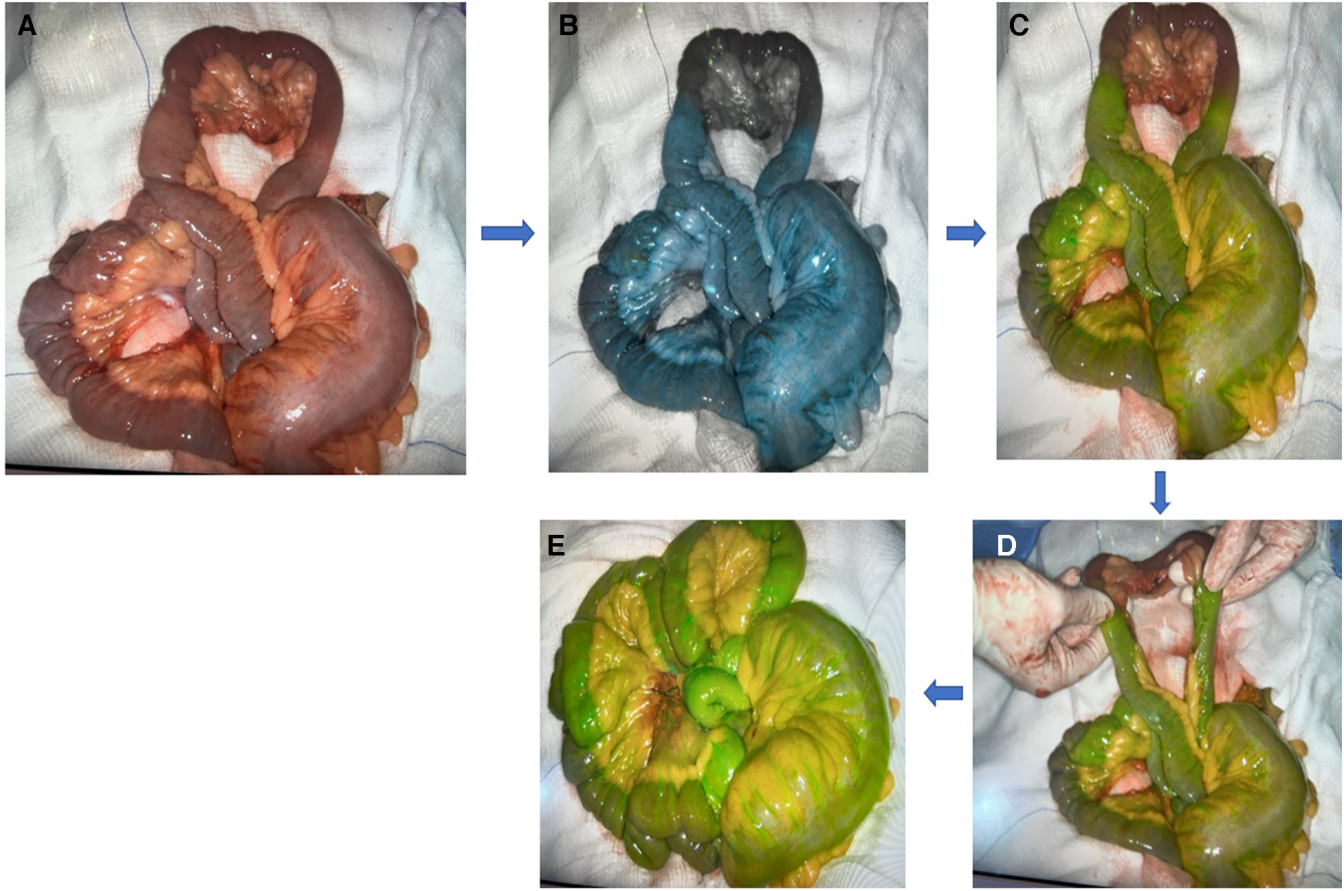
Example of ICG in mesenteric injury: (**A**) mesenteric injury; (**B,C**) blue and green color indicate viable tissue while black color indicates ischemic tissue; (**D**) ICG indicate the limit to resect; (**E**) well-perfused tissue (green color) post resection and anastomosis.

A case series that looked at the use of FA to assess bowel viability in 93 patients in the emergency general surgery setting due to various etiologies, including but not limited to mesenteric ischemia, bowel strangulation, and volvulus, showed that FA changed the management in 29% of the patients. Resection was avoided in four patients. And in 21 (23%) patients, extra bowel length was preserved, although three of them developed further ischemia. FA did also warrant further resection in six patients (87). Further studies have shown similar outcomes (88–92). Figure 5 shows an example of ICG in mesenteric injury.

In patients with incarcerated hernia, Ahmed et al. used ICG to assess bowel viability after reduction. In two of their five patients, bowel viability appeared questionable. IV ICG was used to evaluate the bowel, and resection was avoided. Furthermore, in one patient who had a strangulated hernia, ICG was used to assess the perfusion of the distal and proximal ends before anastomosis. This resulted in further resection to obtain adequately perfused distal and proximal bowel ends prior to the anastomosis (35).

ICG FA has also been used to assess the base of the appendix before appendectomy. Zorzetti et al. reported five cases where ICG was used to assess the base of the appendix prior to appendectomy and the perfusion of the stump after completion of the appendectomy (93).

ICG use is not only limited to the small bowel and colon. ICG can also be used with the esophagus. Imaoka et al. reported on two cases of esophageal stricture after acute esophageal necrosis. The patients did not respond to esophageal dilatation, so they were optimized for planned subtotal esophagectomies. After mobilization of the affected segment of the esophagus, ICG was used to determine the proximal resection margin. Both patients did well with no post-operative leaks (94).

A further use of ICG is to identify minor bowel bleeding. Kawachi et al. detailed a series of patients who had massive, minor bowel bleeding. After appropriate investigation, the patients were taken to a hybrid operating room, and a microcatheter was placed as close as possible to the bleeding site. After laparotomy, ICG was injected through the microcatheter, and the bleeding segment of the bowel was identified grossly without the use of fluorescence imaging, followed by resection of the affected segment (95).

### 3.2 Identification

Patients with small bowel obstruction can have grossly ischemic bowel, which requires resection, and they can have doubtful signs of viability, which may manifest as discoloration, peristalsis, lack of palpable mesenteric pulsation, or intra-abdominal sanguineous fluid. In 1981, Bulkley et al. identified patterns and intensity of sodium fluorescein fluorescence as it pertained to predicted bowel viability (96). More recently, Guerra et al. reported seven patients where ICG FA was used during laparoscopic surgery for small bowel obstruction. In a modified version of the initial effort of Bulkley et al. a modified fluorescence pattern and management was outlined. If the pattern is hyperemic/normal, with increased or normal texture, it is recommended to preserve the bowel segment. According to various patterns and textures, resection was either recommended or not (96, 97).

To quantify the degree of hypoperfusion, the SPY-Q (SPY EliteTM Pack and SPY Eli- teTM Kit, LifeCell Corporation, NJ, USA) imaging system can quantify the intensity of the infrared image emitted by ICG. Foppa et al. described two patients, both with bowel obstruction where ICG was used in an emergency setting surgery and ICG was useful in assessing the bowel perfusion. In both patients, a decision was made not to resect based on the appearance in conjunction with numerical values displayed by the imaging system (98).

ICG fluorescent angiography in the setting of bowel perfusion is easy to perform but subjective in assessment. Studies on porcine models are being conducted to quantify image intensity as it correlates to the degree of hypoperfusion (99). Compared to palpation of pulsation and intestinal temperature in open surgery, ICG use alone in laparoscopic surgery was deemed a viable alternative in a series published of 38 patients with significantly less blood loss and post-operative complications (100).

ICG shows a promising application in assessing intestinal perfusion in emergency general surgery. It can be easily used, provided the setup is available in the open and laparoscopic setting. ICG can be used to evaluate resection lines, anastomotic margins, and bowel viability guided by gross examination. It can also potentially predict areas that may be liable to future ischemia. While it has not been associated with significantly improved outcomes in the acute mesenteric ischemia population, these patients are very ill when they make it to the operating room. However, it can be a guide to more conservative or aggressive resections, which may improve short and long-term outcomes, reduce costs associated with open abdomen and prolonged intensive care stay, and minimize the need for second-look surgery. The drawback is the lack of objectivity and dependence on user interpretation, which may misguide surgeons, leading to bowel necrosis or perforation. Operators must understand the drawbacks of this technology. Accordingly, further studies are needed on outcomes when using ICG compared to gross examination and the possibility of quantifying image intensity as it correlates with pathological examination. Studies on the use of ICG in elective esophageal surgery need to be conducted and carrying that forward to emergency esophageal surgery is required to develop the ICG application further.

## 4 Pancreas

A less commonly reported use of ICG in the hepato-pancreatico-biliary system is for delineating necrotic tissue in the debridement of pancreatic necrosis. Brito-Carmona et al. combined multiple adjuncts to aid with their procedure. Initially, they used intravesical gallbladder ICG injection to delineate the anatomy and aid with the dissection of the hepatocytic triangle, followed by intraoperative cholangiography to assess for choledocholithiasis. ICG was then administered intravenously to visualize the gastric blood supply to facilitate transgastric drainage of walled-off pancreatic necrosis (WOPN). A further administration of ICG was used to delineate the remaining viable pancreatic tissue (56). A further video report by Huang et al. demonstrates ICG-guided video-assisted retroperitoneal debridement (VARD) for pancreatic necrosectomy (101).

In cases of pancreatitis, ICG is administered intravenously. In the reported cases, 3 ml ICG was injected intravenously to determine the extent of pancreatic necrosectomy and help identify the border of healthy pancreatic tissue. ICG can be a helpful adjunct in assessing the viability of remnant pancreatic tissue after the debridement of necrotic pancreatic tissue. Although it is not standardized and there is limited data, if the setup is available, it can be a valuable tool to ensure the remnant pancreatic tissue is viable and potentially guides the adequacy (precision) of the debridement.

## 5 Skin, soft tissue, and burns

Determining burn depth is a challenge for burn surgeons. The burn surgeon typically determines burn depth through assessment of tenderness, blister formation, dermis appearance, the color of the wound, and blanching on the application of pressure (102). Amputations can also be challenging, particularly in patients with poor limb perfusion. ICG offers a potential use in assessing burn depth and flap viability. ICG is injected IV with a dosage varying from 0.5 mg/kg to a non-weight-based 5 mg.

### 5.1 Indications

An aid in assessing burn depth is ICG video-angiography. ICG is injected, and the burn wound depth is evaluated. The initially reported trial in 1995 by Sheridan et al. evaluated ten patients and found that ICG was helpful for the accurate identification of full-thickness burns and partial-thickness burns (103). Still, et al. evaluated nine patients with ICG and corroborated their results with biopsies in seven of those patients. ICG evaluation of burns was accurate when compared to the biopsy results (104).

A further study by Kamolz, et al. involving 20 patients, evaluated ICG uptake, steady state distribution and dye clearance. They could classify the characteristics of the superficial partial thickness, deep dermal partial thickness burns, and full thickness burns as they pertain to ICG video-angiography (105). A recent case series showed the variations of blood supply in burn wounds over time in tissues deemed clinically viable compared to areas that required excision and grafting (106).

Wongkietkachorn et al. evaluated the use of ICG with burn wounds. Initially, they assessed 30 burn wound depths in a prospected multicenter, triple-blinded study. ICG angiography was significantly more accurate than clinical assessment, reaching a sensitivity and specificity of 100% (107). They also published a video showing the ICG in determining wound depth, showing the junction between superficial and deep second-degree burns. The junction was marked and guided by ICG angiography aiding excision (108). This was supported by a prospective multicenter double-blinded study, with overall short-term complete wound closure as high as 96.7% using ICG angiography guidance (109).

### 5.2 Role of ICG in vascular disease

ICG angiography is also used to evaluate flap viability following lower limb amputations. Yang et al. studied the use of ICG angiography in thirteen patients undergoing 17 lower limb amputations. They utilized software to evaluate perfusion using absolute and relative perfusion values quantitatively. The absolute relative perfusion is based on a fixed gray scale consistent between patient to patient. The relative perfusion value assigns a “100% flow” point on a patient-to-patient basis and compares all other points in the image relative to the optimal flow point. Relative perfusion values were reliable predictors of flap outcomes. However, absolute perfusion values failed to support any prognostication indicators (110).

Furthermore, Ahmed et al. reported on a patient with chronic limb ischemia. Time to maximal detection of ICG was recorded pre- and post-revascularization. The time to ICG identification improved after successful revascularization. ICG also served to identify the demarcation of an ischemic toe that was planned for amputation in the same patient (35).

### 5.3 Identification

ICG enables assessment of burn depth and skin perfusion. As Kamloz et al. (105) suggested, superficial partial thickness burns show bright and homogenous fluorescence, rapid uptake, constant and high steady-state distribution, and quick clearance, indicating patency of the small vessels of the sub-papillary and dermal plexus. Deep dermal partial thickness burns show darker mottled fluorescence, with slower uptake and constant but less steady-state distribution indicative of partial patency of the dermal plexus. Full-thickness burns show only large and discrete vessels or no signs of fluorescence (105).

ICG angiography and video-angiography offer a valuable tool in the assessment of burn wounds. It can be used to assess the depth of injury and help define the borders of wound excision. In limb amputations, it can provide necessary guidance and prognostication for the viability of the flap. ICG use in burns and amputations has not been standardized but can be considered for use on a case-by-case basis. Further studies are needed to standardize the use of ICG in burn wounds and amputations. Another potential use in patients with necrotizing fasciitis is to determine the extent of debridement; however, the data is limited and requires further studies on a larger population.

## 6 Less common uses of ICG

### 6.1 Gonads

Testicular and ovarian torsion are medical emergencies. Testicular torsion warrants surgical exploration, detorsion, and possibly orchiectomy. Ovarian torsion requires exploration detorsion and possible oophorectomy.

A prospective report by Nicholson et al. demonstrated the feasibility of using ICG angiography to evaluate ovarian perfusion after detorsion. Twelve patients were diagnosed with ovarian torsion. Nine patients demonstrated entire ovarian perfusion after detorsion, and one patient had partial perfusion. Two patients showed no ICG angiographic perfusion, and post-oophorectomy necrosis was confirmed in one patient. The time interval from injection of the dye to visualization of perfusion was approximately one minute (111).

Similarly, Shirasaki et al. demonstrated testicular salvage with the aid of ICG. A male patient with testicular pain was explored for testicular torsion. The testis had stagnant blood flow and was dark colored. Perfusion was assessed after detorsion with good perfusion, and the testis was preserved with the unremarkable post-operative course, and long-term follow-up (112). Savioe–White et al. demonstrated the use of ICG to determine testicular viability after detorsion; in their case, it required orchiectomy (113).

ICG administered intravenously with the recommendation of 2–5 ml (2.5 mg/ml) can be used with perfusion of the visceral organs. It is used to visualize testicular or ovarian perfusion. Further studies are needed on a larger population of patients. However, ICG angiography is initially useful in assessing gonadal perfusion after detorsion and can potentially affect the operative decision. A summary of pertaining literature on perfusion assessment using ICG is provided in Table 2 (35, 56, 80–92, 94, 95, 97, 100, 101, 103–115).

**Table 2:** Summary of literature pertaining to perfusion assessment using ICG^a^.

Authors	Target Organ	Patients *N*	Urgent care patients *N*	ICG Dose or concentration
Miyashita, et al. (86)	Bowel	1	1	5 mg
Ishiyama, et al. (82)	Bowel	40	40	NR
Joosten, et al. (87)	Bowel	93	93	NR
Nohara, et al. (89)	Bowel	2	2	2.5 mg
Furusawa,et al. (81)	Bowel	1	1	7.5 mg
Ahmed, et al. (35)	Bowel	11	5	7.5 mg
Ryu, et al. (100)	Bowel	38	38 (16)	
Szoka, et al. (90)	Bowel	1	1	2 ml
Guerra, et al. (97)	Bowel	71	71 (7)	2 ml (2.5 mg/ml) repeated as needed
Imaoka, et al. (94)	Bowel	2	2	2.5
Nakamoto, et al. (84)	Bowel	1	1	10 mg
Kawachi, et al. (95)	Bowel	8	8	NR
Аlexander, et al. (91)	Bowel	1	1	0.25 mg
Karampinis, et al. (92)	Bowel	52	52	7.5 mg
Alemanno, et al. (80)	Bowel	1	1	2.5 mg/ml
Zorzetti, et al. (93)	Bowel	5	5	12.5mg
Boni, et al. (114)	Bowel	107	4	0.2 mg/kg × 2
Nowak, et al. (88)	Bowel	4	4	0.2 mg/kg
Nitori, et al. (83)	Bowel	1	1	0.5 mg/kg
Iinuma, et al. (85)	Bowel	1	1	2.5 mg/ml
Huang, et al. (101)	Pancreas	1	1	–
Brito-Carmona, et al. (56)	Pancreas	1	1	3 ml × 2
Ahmed, et al. (35)	Skin, wounds, burns	1	1	7.5 mg
Wongkietkachorn, et al. (109)	Skin, wounds, burns	30 wounds	30 wounds	0.5 mg/kg
Wongkietkachorn, et al. (108)	Skin, wounds, burns	1	1	0.5 mg/kg
Wongkietkachorn, et al. (107)	Skin, wounds, burns	30	30	0.5 mg/kg
Yang, et al. (110)	Skin, wounds, burns	13	13	10 mg
Dissanaike, et al. (106)	Skin, wounds, burns	3	3	5 mg
Kamolz, et al. (105)	Skin, wounds, burns	20	20	0.2 mg/kg
Still, et al. (104)	Skin, wounds, burns	9	9	0.1 mg/kg
Sheridan, et al. (103)	Skin, wounds, burns	10	10	0.2 mg/kg
Klar, et al. (115)	Ovaries	1	1	5 mg
Nicholson, et al. (111)	Ovaries	12	12	–
Savoie-White, et al. (113)	Testes	1	1	2 mg
Shirasaki, et al. (112)s	Testes	1	1	10 mg

^a^ Route of administration was intravenous.

## 7 Conclusions

ICG is a useful adjunct available for emergency general surgeons for varying purposes. Our review and classification of uses for: “anatomical mapping” and “perfusion assessment” can be a helpful schematic for the uses of ICG. The initial hurdle is ensuring the ICG dye and necessary fluorescent imaging camera/scope are available in the operating room. Once set up, ICG is easy to administer and use. While the interpretation to assess the perfusion remains subjective, the future shows promising software development that will offer a more objective interpretation. ICG is a good tool to have in the back of surgeons’ minds; it can be selectively used to complement intraoperative decision-making.

Further studies are needed to evaluate the sensitivity and specificity of ICG use for perfusion assessment. A clear area that needs further development is the subjectivity of ICG in perfusion assessment. Software development to quantify the ICG fluorescent signal strength may increase the accuracy and expand the indications of ICG in the surgical subspecialties.
